# Recent Studies of Rat Ultrasonic Vocalizations—Editorial

**DOI:** 10.3390/brainsci11111390

**Published:** 2021-10-23

**Authors:** Stefan M. Brudzynski, Jeffrey S. Burgdorf

**Affiliations:** 1 Department of Psychology, Brock University, St. Catharines, ON L2S 3A1, Canada; 2 Falk Center for Molecular Therapeutics, McCormick School of Engineering and Applied Sciences, Northwestern University, Evanston, IL 60201, USA; j-burgdorf@northwestern.edu

Since the realization that human emotional experiences and behavior evolved from mammalian ancestors and are evolutionary continuations of animal emotional behavior [1], there has been no clear subdiscipline focusing on the phenomenon of emotion in animals. Although many common limbic structures were identified that were responsible for control of emotional processes in animals and humans [2,3], progress was impeded by a widespread criticism that there is no linguistic means in animals to communicate their emotional states, and thus, researchers cannot know animals’ feelings. However, after extensive studies in the field of behavioral neuroscience for more than 50 years using numerous techniques, such as permanent or reversible brain lesions, electrical or chemical brain stimulation, and behavioral techniques (e.g., place preference, self-stimulation, switch-off behavior, approach and avoidance tests, etc.), the discipline of affective neuroscience was justified and finally defined in 1990s [4,5,6].

The cornerstone of affective neuroscience studies relates to brain mechanisms of emission of animal vocalizations because they convey animal emotional arousal. Animal calls are honest advertisement of their affective and motivated states and were used as measures of these states. The most studies involved rodent ultrasonic vocalizations because they express affective arousal better than many other experimental animals and are convenient for pharmacological studies. This was demonstrated in the first report of pharmacological induction of the emission of rat 22 kHz ultrasonic vocalizations by direct intracerebral injection of carbachol, a drug mimicking the action of acetylcholine [7]. The field of studies of ultrasonic vocalizations started rapidly growing and led to the identification and validation of two types of rat ultrasonic calls that signal two different emotional states with opposite valences. Emission of 22 kHz calls signals aversive arousal and a negative state, and emission of 50 kHz calls signals appetitive arousal and a positive state [8].

Pharmacological and behavioral studies of vocalizations enabled researchers to identify the brain systems responsible for aversive/punishing and appetitive/rewarding states. The valence-specific calls provide an invaluable insight into the rodent emotional processes and expression of their basic internal states. Although the emission of animal vocalizations presents a phylogenetically older and simpler communication system than human speech, it reliably serves animals as an overt announcement of their emotional arousal, and its valence is highly adaptive. Vocal expression of emotional arousal in animals is a direct homolog to expression of emotional states by human vocalizations such as crying or laughing [9,10].

Interest in emission of rodent ultrasonic vocalizations and their biological functions has markedly increased in recent years, particularly their use in animal models of neuropsychiatric diseases and disorders. Alteration in emission of ultrasonic vocalizations appeared to be a sensitive index of many pathologies. The present Special Issue offers a collection of recent studies on rats using recording and analysis of ultrasonic vocalizations as a guide to animals’ emotional arousal and emotional states. This is particularly useful in modelling numerous human neurodevelopmental and neuropsychiatric disorders and diseases.

The publications in the Special Issue are not representing predetermined topics but rather serve as examples of many approaches demonstrating different studies of ultrasonic vocalizations and different ways of modelling psychopathological states from infancy to adulthood.

The opening article of the Special Issue thoroughly reviews the socio-physiological functions of ultrasonic communication in rats, as well as the brain mechanisms of emotional arousal, and discusses how emission of these calls should be interpreted. Examples of these interpretations are presented in the following articles.

Studies of the emission of distress vocalizations in infant rats caused by maternal separation demonstrated their sensitivity to levels of vasopressin having anxiogenic effect. Application of angiotensin antagonists decreased emission of pup distress ultrasonic calls (D. Zelena laboratory). Pharmacological activation of the maternal immune system also had an effect on the emission of ultrasonic vocalizations of the offspring and impaired socio-communicative functioning of adolescent rats (A. Nikiforuk laboratory). Consumption of alcohol by pregnant rats has been known to have detrimental effects on pups’ development, which was reflected in their ultrasonic distress calls. However, lactational exposure of a mother to alcohol appeared to have more devastating effect on pups and their distress calls than gestational exposure to alcohol (M.A. Shahrier laboratory).

Emission of ultrasonic vocalizations is coordinated with respiration rate, and call emission and respiration rate collectively affect brain oscillatory activities (A-M. Mouly laboratory). Sex differences in emission of ultrasonic vocalizations were also reviewed and showed differences not only in structures of the rat larynx but also in the acoustic parameters of the vocalizations between sexes (M.R. Ciucci laboratory).

In a couple of articles, emissions of adult ultrasonic vocalizations were studied in stressful situations as observation fear learning, which is associated with social transmission of the demonstrator’s emotional state and induction of an empathy-like or anxiety state in the observer rat. Although fear learning was more pronounced in rats with higher trait anxiety, it appeared in this study that communication by ultrasonic vocalizations was not critical for observational fear learning (M. Fendt laboratory). However, if a rat (observer) was witnessing other rats receiving foot shock (demonstrator), the emission of ultrasonic calls was dependent on an earlier experience of these rats with the foot shock and on the presence of a warning sound before each foot shock. Particularly the warned rats, in addition to typical, long 22 kHz vocalizations, emitted short 22 kHz calls (less than 100 ms in duration), and in warned pairs with a naïve observer, 22 kHz calls were intermixed with the emission of 50 kHz vocalizations, which suggested vocal social buffering (A. Hamed laboratory). Earlier experience with foot shocks also had an influence on the subsequent responses of rats to playback of 50 kHz or 22 kHz vocalizations. Initial foot shock experience caused that rats vocalized more often with longer duration calls and a higher sound frequency than the control animals; these rats also showed a lower heart rate, higher locomotor activity in response to playback of 50 kHz vocalizations, and decreased their activity following playback of 22 kHz calls (R. Filipkowski laboratory).

Ultrasonic vocalization responses in tickling tests (heterospecific play of juvenile rats with human hand) showed that rats could be divided into two groups having high or low positive affectivity. It was shown that dopamine in the nucleus accumbens regulated responses to stress in different way in these groups. Stress increased the release of dopamine in rats with high positive affectivity but decreased it in the low positive affectivity rats (J. Harro laboratory). In another study, rats were subjected to a tickling procedure and then tested for novel object recognition and for the alternation behavior in a Y-maze. Rats emitted 50 kHz vocalizations but not 22 kHz calls during these tests; however, responses to novel objects were impaired if the animals consistently emitted 22 kHz in the initial tickling tests (N. Simola laboratory).

Emission of ultrasonic vocalizations recorded in rats awaiting play partner or food reward showed that emission of trill type calls of 50 kHz was increased by play reward but not by food reward (S.M. Pellis and D.R. Euston laboratories). In another experiment, using chemogenetic reversible inhibition of the nucleus accumbens neurons suppressed the emission of trill 50 kHz calls without changes in emission of the flat (unmodulated) 50 kHz vocalizations or other vocalizations (S.V. Mahler laboratory).

Publications contained within this Special Issue are also further demonstrating the use of the emission of ultrasonic vocalizations in modelling many neuropsychiatric dysfunctions and diseases, such as Parkinson’s disease, affective disorders, autism, addiction, developmental abnormalities, and many other pathologies. This is demonstrated in a 6-hydroxydopmine model of Parkinson’s disease (M.R. Ciucci laboratory); autistic spectrum disorder, modelled by viral-like activation of the maternal immune system during pregnancy, which may affect the neurodevelopment of offspring (A. Nikiforuk laboratory); in models of posttraumatic stress disorder (R. Filipkowski laboratory); and in studies of the mechanisms of drug addiction, particularly with self-administration of the powerful opioid drug fentanyl (M.O. West laboratory). Other approaches may target many neuropsychiatric disorders together, as studied using the cross-disorder risk gene CACNA1C, which is strongly associated with multiple neuropsychiatric dysfunctions. Using Cacna1c haploinsufficiency in rats, robust deficits in socio-affective communication through 22 kHz and 50 kHz vocalizations and associated alterations in social behavior were found (M. Wöhr laboratory).

Analysis of recorded vocalizations is time consuming and subjected to numerous errors given the researcher’s experience. A web-based automated ultrasonic vocalization scoring tool, called Acoustilytix, is described. This system implements a machine learning methodology in the detection of ultrasonic calls and their classification, which is not related to the environment or the scorer’s experience. It uses automated learning principles without the need for an expert to be present (Ch.L. Duvauchelle laboratory).

This Special Issue is a useful update on the current research directions using recordings of ultrasonic vocalizations and on the different methodological approaches.
